# Inactivation of Venezuelan Equine Encephalitis Virus Genome Using Two Methods

**DOI:** 10.3390/v14020272

**Published:** 2022-01-28

**Authors:** Mahgol Behnia, Alan Baer, Andrew M. Skidmore, Caitlin W. Lehman, Nicole Bracci, Kylene Kehn-Hall, Steven B. Bradfute

**Affiliations:** 1 Department of Internal Medicine, University of New Mexico Health Sciences Center, Albuquerque, NM 87131, USA; mabehnia@salud.unm.edu (M.B.); amskidmore@salud.unm.edu (A.M.S.); 2 National Center for Biodefence and Infectious Diseases, George Mason University, Manassas, VA 20110, USA; alangbaer@gmail.com (A.B.); kkehnhall@vt.edu (K.K.-H.); 3 Department of Biomedical Sciences and Pathobiology, Virginia-Maryland College of Veterinary Medicine, Virginia Tech, Blacksburg, VA 24061, USA; woodsonc@vt.edu (C.W.L.); nbracci@vt.edu (N.B.)

**Keywords:** encephalitis, Venezuelan equine encephalitis virus, RNA fragmentation, cDNA synthesis, viral genome inactivation

## Abstract

Venezuelan equine encephalitis virus (VEEV) is an Alphavirus in the Togaviridae family of positive-strand RNA viruses. The viral genome of positive-strand RNA viruses is infectious, as it produces infectious virus upon introduction into a cell. VEEV is a select agent and samples containing viral RNA are subject to additional regulations due to their infectious nature. Therefore, RNA isolated from cells infected with BSL-3 select agent strains of VEEV or other positive-strand viruses must be inactivated before removal from high-containment laboratories. In this study, we tested the inactivation of the viral genome after RNA fragmentation or cDNA synthesis, using the Trinidad Donkey and TC-83 strains of VEEV. We successfully inactivated VEEV genomic RNA utilizing these two protocols. Our cDNA synthesis method also inactivated the genomic RNA of eastern and western equine encephalitis viruses (EEEV and WEEV). We also tested whether the purified VEEV genomic RNA can produce infectious virions in the absence of transfection. Our result showed the inability of the viral genome to cause infection without being transfected into the cells. Overall, this work introduces RNA fragmentation and cDNA synthesis as reliable methods for the inactivation of samples containing the genomes of positive-strand RNA viruses.

## 1. Introduction

RNA viruses are grouped based on the types of RNA that serve as their genome. The genome of a positive-strand RNA virus has mRNA characteristics and can be used as a template for the production of infectious virions upon introduction into susceptible cells [1]. Among the highly pathogenic positive-strand RNA viruses that infect humans, some Flaviviruses and Alphaviruses are categorized as bioterrorism agents because of their infectivity in aerosolized form and ability to cause severe debilitating diseases in humans and livestock. For example, the Far Eastern subtype of Tick-borne encephalitis virus, a Flavivirus, and eastern equine encephalitic virus (EEEV), an Alphavirus, cause encephalitis in humans with high mortality rates and are select agents [2,3,4]. Currently, many laboratories use modern technologies such as RNA sequencing and qRT-PCR to study positive-strand RNA viruses and virus–host interactions. Many of these techniques rely upon RNA extraction, manipulation, and transfer to lower containment facilities for downstream analysis. The ability of the positive-strand RNA genome to initiate the viral life cycle upon introduction to the susceptible cells raises the concern about the production of the highly pathogenic positive-strand select agents in unauthorized facilities. This is not a concern in select agents with a negative-strand RNA genome, as the negative-strand RNA is not a template for translation [1]. Thus, to prevent the mass production of positive-strand select agents in unauthorized laboratories, the Center for Disease Control (CDC) and the United States Department of Agriculture (USDA) provided guidelines outlining the proper regulation of the select agent positive-strand RNA genome before downstream manipulations [5].

Venezuelan equine encephalitis virus (VEEV) is an enveloped, positive-strand RNA virus from the Alphavirus genus in the Togaviridae family. The VEEV complex is a group of seven species containing fourteen antigenic varieties. The VEEV species consists of four serotypes named IA/B, IC, ID, and IE, all of which cause disease in humans. VEEV serotypes can have enzootic or epizootic life cycles. The VEEV enzootic strains ID and IE cause endemics in many different species of mammals. The epizootic strains IA/B and IC cause severe disease with a high mortality rate in equines and are responsible for VEEV outbreaks in horses and humans [6,7,8]. Although typically spread via mosquitos, VEEV is also highly infectious through the aerosol route and a potential bioterrorism agent that causes severe encephalitis in humans [7]. Only epizootic strains of VEEV are categorized as bioterrorism agents. For example, VEEV Trinidad donkey (TrD) is a biosafety level 3 (BSL-3) agent known as a bioterrorism agent. Currently, there is no US FDA-approved VEEV vaccine available for humans. Only a live attenuated vaccine (VEEV TC-83) is available for at-risk personnel. Given that VEEV TrD is a select agent with a positive-strand RNA genome, the inactivation of the purified VEEV genome or RNA from VEEV infected cells is required before transfer to lower containment facilities.

We utilized VEEV infected cells to analyze different methods for viral RNA inactivation for downstream RNA sequencing and qRT-PCR and to assess the ability of viral RNA to produce infectious virus in the absence of transfection into cells. We found that RNA fragmentation or cDNA synthesis followed by RNase treatment successfully inactivated VEEV RNA extracted from the infected cells. Likewise, cDNA synthesis followed by RNase treatment also inactivated genomic RNA from related encephalitic alphaviruses, EEEV and western equine encephalitis virus (WEEV). We also found that co-incubation of RNA from VEEV infected cells was not sufficient to induce the production of infectious virions, showing that direct introduction into cells via transfection or other methods is required for this to occur.

## 2. Materials and Methods

Cell culture: three batches of primary mouse cortical neurons (Gibco, Grand Island, NY, USA, Cat# A15586) were seeded in 6-well plates (10^6^ cells/well) precoated with 25 μg/mL of poly-D-lysine four days before infection. The cells were maintained in neurobasal media (Gibco, Cat# 21103) supplemented with 0.5 mM GlutaMAX-I (Gibco, Cat# 35050) and 2% (*v*/*v*) B-27 (Gibco, Cat# 17504) at 37 °C and 5% CO_2_ until infection. Vero E6 cells (ATCC, Manassas, VA, USA, Cat# CRL-1586) were cultured at a density of 0.3 × 10^6^ cells/well in 6-well plates, 0.1 × 10^6^ cells/well or 0.3 × 10^6^ cells/well in 12-well plates, and 1.4 × 10^4^ cells/well in 96-well plates maintained at 37 °C, and 5% CO_2_ in Dulbecco’s Modified Eagle Medium (DMEM) supplemented with 10% fetal bovine serum (FBS) and 1% Pen Strep Glutamine (100×) (Gibco, Cat#10378016).

Viral stock preparation: Trinidad donkey (TrD) and the live attenuated vaccine (TC-83) strains of Venezuelan equine encephalitis virus were received from the Biodefense and Emerging Infections Research Resources Repository (BEI Resources), NR-332 and NR-63. The viral stocks were expanded in Vero E6 cells and quantitated using plaque assay. The EEEV GA97 strain was provided by Dr. Jonathan Jacobs (MRIGlobal) and the WEEV 1930 California strain (VR-70) was purchased from ATCC. EEEV and WEEV viral stocks were expanded in Vero cells (ATCC, Cat#CCL-81) and quantitated via plaque assay.

Viral infection: at day four post-seeding, primary mouse cortical neurons were infected with a multiplicity of infection (MOI) of 5 of VEEV TrD or TC-83. The viral stocks diluted in minimum essential medium, and added to the cells after aspirating 1 mL of media from each well of 6-well plate. The plates were incubated at 37 °C and 5% CO_2_ for 2 h with rocking every 30 min. After two hours, 0.5 mL of media aspirated from each well followed by 1.5 mL of complete pre-warmed media added to each well. Twenty-four hours post-seeding, Vero E6 cells cultured at the density of 0.3 × 10^6^ cells were infected with 5 MOI of VEEV TC-83 as explained previously.

Total RNA isolation: total RNA was isolated from control and infected mouse neurons at 0, 16, and 24 h post-infection (p.i.) using a Direct-zol RNA Microprep kit (Zymo Research, Irvine, CA, USA, Cat# R2062) according to the manufacturer’s protocol. All the steps were conducted at room temperature, and columns were centrifuged at 15,700× *g* for 30 s unless otherwise specified. To isolate RNA, media was aspirated from each well, and the cells were lysed in 500 μL of TRI reagent (Zymo Research, Irvine, CA, USA, Cat# R2050-1-50). The cell lysates were transferred into 1.5 mL microcentrifuge tubes, vortexed at maximum speed for 10 s, and incubated at room temperature for five minutes. After the incubation period was completed, the cell lysate was mixed with an equal volume of 100% ethanol, and the mixture was transferred into a Zymo-Spin™ IC Column2, followed by centrifugation. Four hundred ul of RNA wash buffer was added to each column and centrifuged to remove any contamination. Each washed column was treated with a mixture of 5 μL DNase I (6 U/μL) and 35 μL DNA Digestion Buffer for 15 min and washed twice with 400 μL of RNA prewash. The columns were then washed by adding 700 μL of RNA wash buffer followed by centrifugation for 1 min. A total of 10 μL of RNase-free water was added directly to each column, and the columns were centrifuged for 1 min to elute RNA from the columns. The extracted RNAs were stored at −80 °C in a freezer.

Total RNA was extracted from uninfected and TC-83 infected Vero E6 cells at 24 h post-infection using a Direct-zol RNA Microprep kit (Zymo Research, Cat# R2062) according to the manufacturer’s protocol.

For experiments analyzing entry of RNA in the absence of transfection, total RNA was extracted from Vero E6 cells that were incubated with 10 μg of VEEV TC-83 RNA or Vero E6 RNA (control) in their media at 72 h post-treatment using a Direct-zol RNA Miniprep kit (Zymo Research, Cat#2050) according to the manufacturer’s protocol.

Viral RNA infectivity test: to test the ability of the viral RNA to start the infection without being introduced to cells through lipid-based transfection, we cultured Vero E6 cells at a concentration of 1.4 × 10^4^ cells/well in a 96-well plate and added 1 μg of total RNA from VEEV TC-83 infected Vero E6 cells to the media in each well. The experiment was done in triplicate. One μg/well of total RNA, unfragmented RNA extracted from TC-83 infected Vero E6 cells, was transfected to Vero E6 cells as a positive control. As negative controls, 1 μg/well of the RNA from uninfected Vero E6 cells was directly added or transfected to cells using TransIT-mRNA transfection kit (Mirus Bio LCC, Madison, WI, USA, Cat# Mir2225) to Vero E6 cells in triplicates. The cells were maintained at 37 °C and 5% CO_2_, and pictures were taken 72 h, 7 d, and 13 d after adding the RNA to the cells. To confirm the lack of viral RNA inside the cells incubated with viral RNA in their media in the absence of transfection, we cultured Vero E6 cells at the concentration of 0.1 × 10^6^ cells/well in 12-well plates and added 10 μg total RNA from VEEV TC-83 infected Vero E6 cells per well to the media in triplicates. As a negative control, we added 10 μg of total RNA from uninfected Vero E6 cells to the media of each well in triplicate. The cells were maintained at 37 °C and 5% CO_2_ for 72 h. At 72 h post-treatment, the media was aspirated from all the wells, and the cells were washed with 1× PBS. To eliminate the viral RNA outside the cells, the cells in each well were treated with one unit of RNase A (Millipore Sigma, St. Louis, MO, USA, Cat# 10109142001) in 1× PBS for 5 min. The cells were then washed with 1× PBS twice, followed by Trypsinzing and pelleting by centrifugation at 800× *g* for 5 min. The cell pellets were flash frozen and the extracted RNA was used for cDNA synthesis and qPCR.

qRT-PCR: RNAs extracted from the Vero E6 cells (treated by adding 10 μg RNA extracted from VEEV TC-83 infected cells or uninfected Vero E6 cells to their media) were converted to cDNA using the SuperScript II reverse transcriptase kit according to manufacturer’s protocol (Invitrogen, Carlsbad, CA, USA, Cat # 18064022). A total of 500 ng of RNA was used per cDNA synthesis reaction. This cDNA was diluted 1:100 and used in qRT-PCR using the Applied Biosystems TaqMan Fast Advanced Master Mix (Cat # 4444557) with a final primer concentration of 0.5 μM for each primer, and a final probe concentration of 0.25 μM for each probe; 2 μL of diluted cDNA was used per reaction. The reaction cycling was carried out according to the manufacturer’s instructions in a QuantStudio5 qRT-PCR machine. An initial 2 min incubation at 50 °C was included. A total of forty cycles were performed. Primers and probes were custom ordered from IDT and based upon previous work (Table 1) [9].

RNA Fragmentation: to inactivate the viral RNA, total RNA extracted from virally infected cells was fragmented using an Ion Total RNA-seq Kit v2 (ThermoFisher Scientific, Waltham, MA, USA, Cat# 4479789) according to the manufacturer’s instructions. The reaction mixture for each RNA sample was assembled on ice in a 0.2 mL PCR tube by mixing total RNA (500 ng), 10× RNase III Reaction Buffer (1 μL), and RNase III (1 μL), and incubated at 37 °C for 10 min. Immediately after incubation, 20 μL of RNase-free water was added to the fragmented RNA, and the tubes were chilled to 4 °C. To purify the fragmented RNA, 5 μL of nucleic acid-binding magnetic beads (provided in Ion Total RNA-seq Kit v2) was added to each well on the processing plate and mixed with 90 μL of Binding Solution Concentrate by pipetting. The fragmented RNA reactions were transferred to bead-containing wells, followed by adding 150 μL of 100% ethanol to each well and mixing. The samples were incubated for 5 min at room temperature to let the fragmented RNA bind to the beads, then the processing plate was placed on the magnetic stand to separate the beads from the solution, and the beads were washed with wash solution for 30 s. To elute the RNA from the magnetic beads, the processing plate was removed from the magnetic stand, and 12 μL of pre-warmed (37 °C) nuclease-free water was added to each sample and incubated for 1 min. The processing plate was placed on the magnetic stand for 1–2 min to separate the beads from the solution, and the eluent was transferred into a new low bind tube.

Quantification of the fragmented RNA: the Fragmented RNAs were quantified using a Qubit RNA HS assay kit (Invitrogen, Carlsbad, CA, USA, Cat#Q32852), and on a Qubit 4 Fluorometer (ThermoFisher Scientific).

Transfection: Vero E6 cells were seeded at a density of 1.4 × 10^4^ cells/well in a 96-well plate 24 h before transfection. Twenty-four hours post-seeding, Vero E6 cells were transfected with 50 ng/well of total RNA obtained from VEEV TC-83 or TrD infected cells (positive control), uninfected cells (negative control), and fragmented RNA from VEEV TC-83 or TrD infected cells using a TransIT-mRNA transfection kit (Mirus Bio LLC, Cat# 2225). Three wells were transfected per sample. The cells were checked under the microscope for cytopathic effect (CPE) and the supernatants were collected for plaque assay at 72 h post-transfection.

For determination of assay sensitivity, total RNA was extracted 24 h after VEEV TC-83 infection of Vero E6 cells, diluted, and transfected to Vero E6 cells in different concentrations (50, 5, 0.5, and 0.05 ng/well). The Vero E6 cells were cultured at a concentration of 1.4 × 10^4^ cells/well in a 96-well plate, and four wells per RNA sample were transfected. As a negative control, total RNA obtained from uninfected Vero E6 was diluted to similar concentrations as the RNA from the VEEV TC-83 infected cells and transfected to Vero E6 cells using a TransIT-mRNA transfection kit (Mirus Bio LLC, Cat# 2225). The cells were assessed under the microscope for cytopathic effect, and images were taken 72 h post-transfection.

Plaque assay: Vero E6 cells were seeded at a density of 0.3 × 10^6^ cells/well in 12-well plates 24 h before infection. The cells were incubated with ten-fold serial dilutions of supernatants from cells transfected with fragmented or unfragmented (control) VEEV TC-83 RNA for one hour at 37 °C with rocking at 30 min. Dilutions 10^−1^ through 10^−7^ of the supernatants from cells transfected with fragmented VEEV TC-83 RNA and dilutions 10^−4^ through 10^−10^ of the supernatant from cells transfected with unfragmented VEEV TC-83 RNA were used in the plaque assay. After the incubation period, the inoculum was removed, one ml of 1% agarose in Modified Eagle Medium supplemented with 2.5% FBS was added to each well, and the plates were incubated at 37 °C and 5% CO_2_ for 72 h. For fixing, 1 mL of 4% formaldehyde was placed on top of the agar overlay and incubated at 4 °C overnight. After the incubation period, the fixed monolayer was stained with 0.8% crystal violet solution, the plaques were quantified, and virus concentrations were recorded as PFU/mL. Three wells per dilution were tested in two separate experiments. 

cDNA synthesis, RNase Digestion, and cDNA transfection: purified RNA (2 μg) was converted to cDNA using a high-capacity RNA-to-cDNA kit (ThermoFisher Scientific, Waltham, MA, USA, Cat# 4387406) according to the manufacturer’s instructions. For the RNase treatment, RNase A 100 mg/mL (Qiagen, Germantown, MD, USA, Cat # 19101) was first diluted to make a 1 mg/mL working stock. Next, 1 μL of the RNase A (1 mg/mL working stock) and 1 μL (10 U/μL) of RNase H (ThermoFisher Scientific, Cat #AM2293) were added to each sample. Samples were incubated at 37 °C for 20 min. The entire cDNA preparation (20 μL) was transfected into a T75 flask of Vero cells using the Attractene transfection reagent (Qiagen, Germantown, MD, USA, Cat# 301005), according to the manufacturer’s instructions. Cells were assayed for CPE via microscopy at Day 4. Following CPE observations at Day 4, five ml of media supernatant was removed and transferred to a fresh set of cells. Five ml of fresh media was also added to each flask to ensure that enough growth factors are present to allow cell growth. Cells were assayed for CPE via microscopy again at Day 7. The Day 7 supernatants were assayed for infectious virus via plaque assay as previously described [10].

## 3. Results

### 3.1. Fragmentation of VEEV RNA Renders It Non-Infectious

**Figure 1:**
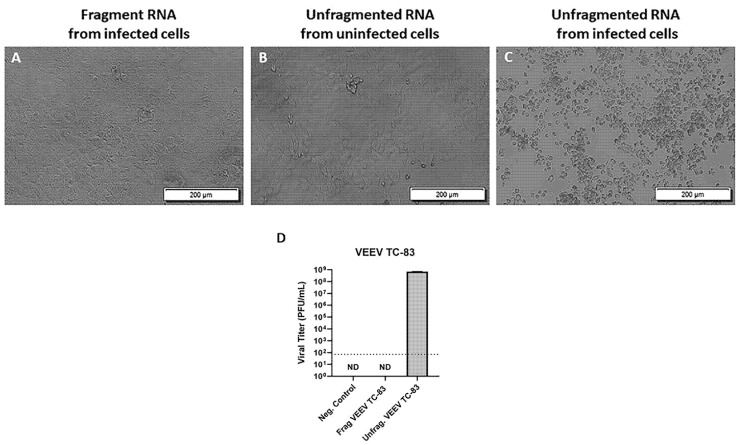
Fragmentation of VEEV TC-83 RNA renders it non-infectious. Vero E6 cells were transfected with total RNA purified from uninfected or TC-83 infected primary mouse neurons at 72 h post-transfection. Three wells/sample transfected; (**A**) Vero E6 cells transfected with 50 ng/well of fragmented total RNA purified from TC-83 infected primary mouse neurons; (**B**) Vero E6 cells transfected with 50 ng/well of total RNA purified from uninfected primary mouse neurons (negative control); (**C**) Vero E6 cells transfected with 50 ng/well of total RNA purified from TC-83 infected primary mouse neurons. The results shown (**A**–**C**) are representatives of three wells per experiment and two separate experiments. (**D**) Viral titers of VEEV TC-83 measured in plaque assays using supernatants collected from cells 72 h after transfection with fragmented VEEV TC-83 RNA or unfragmented VEEV TC-83 RNA (positive control). Uninfected Vero E6 cell supernatants were used as the negative control. ND = none detected. Viral titer reported at 72 h p.i. and as the average of PFU/mL for two separate experiments. The dotted line indicates the limit of detection.

To test whether fragmentation of positive-sense viral RNA inactivates the virus genome and its ability to produce infectious virions, we infected primary mouse neurons with 5 MOI VEEV TC-83 followed by RNA extraction at 0, 16, and 24 h p.i. The extracted RNA from the infected cells was then fragmented and transfected to Vero E6 cells in triplicate. Our results showed no CPE in the Vero E6 cells transfected with the fragmented RNA at 72 h post-transfection (Figure 1A), which was similar to the result from the negative control (cells transfected with unfragmented RNA from uninfected cells) (Figure 1B). In contrast, the cells transfected with unfragmented total RNA purified from VEEV TC-83 infected cells (positive control) showed extensive cell death, suggesting the production of infectious virions (Figure 1C). To further confirm the lack of production of the infectious virion post-fragmentation of viral RNA, we tested supernatants from the cells transfected with the fragmented RNA purified from VEEV TC-83 infected cells and those transfected with the unfragmented RNA from VEEV TC-83 infected cells in a plaque assay. Our result showed the formation of the plaques with an average viral concentration of 7 × 10^8^ PFU/mL in the Vero E6 cells that were incubated with the supernatant from the cells transfected with unfragmented viral RNA (Figure 1D). In contrast, no plaques were observed in the cells incubated with the supernatant from the Vero E6 cells transfected with the fragmented viral RNA. These results suggest the inactivation of the viral RNA post-fragmentation and confirm the fragmented RNA’s inability to produce infectious virions. To ensure that this inactivation did not compromise downstream analysis, RNA sequencing was performed and an increase in mapped reads to the VEEV genome in VEEV infected primary mouse neurons at 16 and 24 h p.i. was detected (data not shown). This increase in the mapped reads to the viral genome confirms that the inactivated RNA was suitable for downstream analysis.

**Figure 2:**
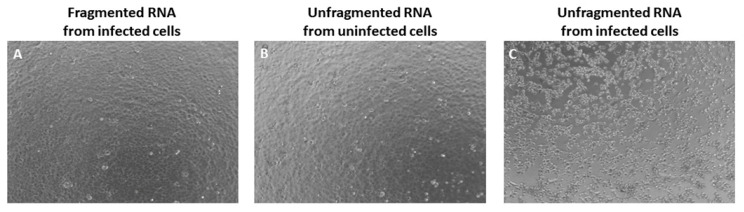
Fragmentation of VEEV TrD RNA renders it non-infectious. Vero E6 cells transfected with total RNA purified from uninfected or TrD infected primary mouse neurons at 72 h post-transfection; (**A**) Vero E6 cells transfected with 50 ng/well of fragmented total RNA purified from TrD infected primary mouse neurons; (**B**) Vero E6 cells transfected with 50 ng/well of total RNA purified from uninfected primary mouse neurons (negative control); (**C**) Vero E6 cells transfected with 50 ng/well of total RNA purified from TrD infected primary mouse neurons. The results shown are representative of three wells per experiments and three separate experiments.

We further confirmed the result from the VEEV (TC-83) vaccine strain using the wild-type Trinidad Donkey (TrD) VEEV genome containing total RNA from primary mouse neurons infected with TrD. Similar to the result from VEEV TC-83, the Vero E6 cells transfected with fragmented RNA from VEEV TrD infected cells and the unfragmented RNA from uninfected cells (negative control) remained as a monolayer without any sign of CPE (Figure 2A,B). In contrast, the cells transfected with unfragmented RNA from VEEV TrD infected cells showed extensive CPE (Figure 2C). Overall, our results suggest the inactivation of the viral genome via fragmentation as a reliable method for positive strand viral RNA inactivation.

### 3.2. Low Concentrations of Unfragmented Viral RNA Causes Cytopathic Effect in Vero E6 Cells

**Figure 3:**
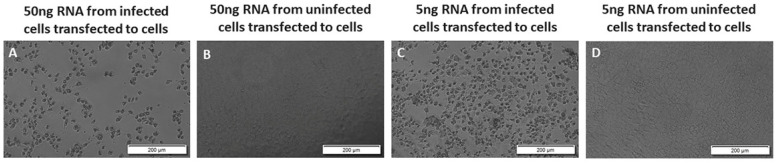
Low concentrations of viral RNA cause cytopathic effect in Vero E6 cells. Total RNA purified from uninfected or TC-83 infected Vero cells transfected to Vero cells in 50 or 5 ng/well, images were taken at 72 h post-transfection. (**A**) 50 ng of total RNA from TC-83 infected Vero cells transfected to Vero cells; (**B**) 50 ng of total RNA from uninfected Vero cells transfected to Vero cells; (**C**) 5 ng of RNA from TC-83 infected Vero cells transfected to Vero cells; (**D**) 5 ng of RNA from uninfected Vero cells transfected to Vero cells. The results shown are representative of three wells in two separate experiments.

**Figure 4:**
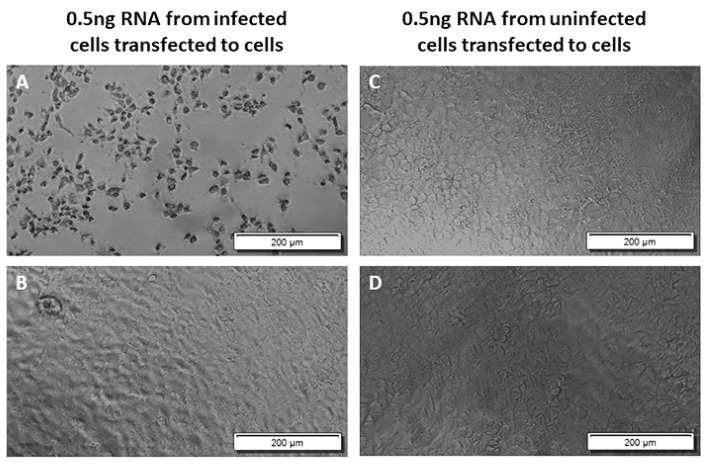
Low concentration of viral RNA causes cytopathic effect in Vero E6 cells. Total RNA purified from uninfected or TC-83 infected Vero E6 cells and transfected into Vero E6 cells in 0.5 ng/well; images were taken at 72 h post-transfection. (**A**,**B**) 0.5 ng of total RNA from TC-83 infected Vero cells transfected to Vero cells; (**C**,**D**) 5 ng of RNA from uninfected Vero cells transfected to Vero cells. The result shown is representative of four wells in two separate experiments.

To find the lowest concentration of viral RNA that can cause CPE after transfection, we transfected different concentrations of total RNA from the VEEV TC-83 infected Vero E6 cells to Vero E6 cells and assessed the cells for CPE at 72 h post-transfection. Our results demonstrated cell death in all the wells transfected with 50 ng/well or 5 ng/well of unfragmented RNA from VEEV TC-83 infected cells (Figure 3). Among four wells transfected with 0.5 ng/well of RNA from VEEV TC-83 infected cells, two showed CPE, and two did not show cell death; similar results were obtained in two separate experiments (Figure 4). Our result shows 5 ng of RNA as the lowest concentration of the RNA that consistently causes CPE, which suggests production of infectious virus after transfection to the cells using the protocol in this report (Figure 4).

### 3.3. Viral RNA Requires Artificial Methods to Enter Cells

**Figure 5:**
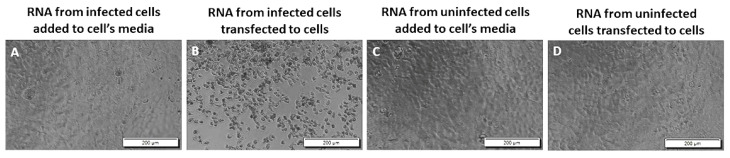
Viral RNA does not cause CPE without artificial entry. Total RNA purified from uninfected or TC-83 infected Vero cells directly added or transfected to Vero cells, images taken at 72 h post-administration; (**A**) 1 μg/well of total RNA from TC-83 infected Vero cells directly added to Vero cells; (**B**) 1 μg/well of RNA from TC-83 infected Vero cells transfected to Vero cells (positive control); (**C**) 1 μg/well of total RNA from uninfected Vero cells directly added to Vero cells (negative control); (**D**) 1 μg/well of total RNA from uninfected Vero cells Transfected to Vero cells (negative control). The result shown is representative of three wells in two separate experiments.

To test whether viral RNA can start the infection in susceptible cells without being introduced to cells through transfection, we directly added 1 μg/well of total RNA extracted from VEEV TC-83 infected Vero E6 cells to Vero E6 cells cultured in 96-well plates in triplicate. Similar to the negative controls, our result did not show any CPE in the cells that received 1 μg of the viral genome containing RNA in their media at 72 h, 7 d, and 13 d post-administration (Figure 5A,C,D, Figure S1 and S2). In contrast, the cells transfected with 1 μg/well of RNA from VEEV TC-83 infected cells resulted in vast cell death (Figure 5B). Our result suggests the inability of the viral genome to induce productive infection without being introduced to the cells.

To further confirm the absence of the viral RNA inside the cells exposed to viral RNA in their media, we added 10 μg of total RNA extracted from VEEV TC-83 infected Vero E6 cells to the media of the Vero E6 cells cultured in 12-well plates in triplicate. As a control, 10 μg of total RNA from uninfected Vero E6 cells were added to the media of the Vero E6 cells in triplicate. The extracted RNA was assayed by qRT-PCR using primers and probes detecting VEEV in the capsid and nonstructural protein 1 (nsP1) sequences. Total RNA extracted from VEEV TC-83 infected Vero E6 cells was used as a positive control for these experiments. The qRT-PCR of the RNA from VEEV TC-83 infected cells detected the viral capsid and nsP1 sequences at average cycle thresholds (Ct) of 11.5 and 16.3, respectively. In contrast, the qRT-PCR did not detect viral nsP1 in the samples from the cells treated by adding viral RNA to their media. The qRT-PCR detected viral capsid sequence at the cycle thresholds >36, demonstrating a low copy number of the viral RNA in the cells treated with viral RNA in their media (Figure S3).

### 3.4. cDNA Synthesis Followed by RNase Treatment Renders VEEV, EEEV and WEEV RNA Non-Infectious

**Figure 6:**
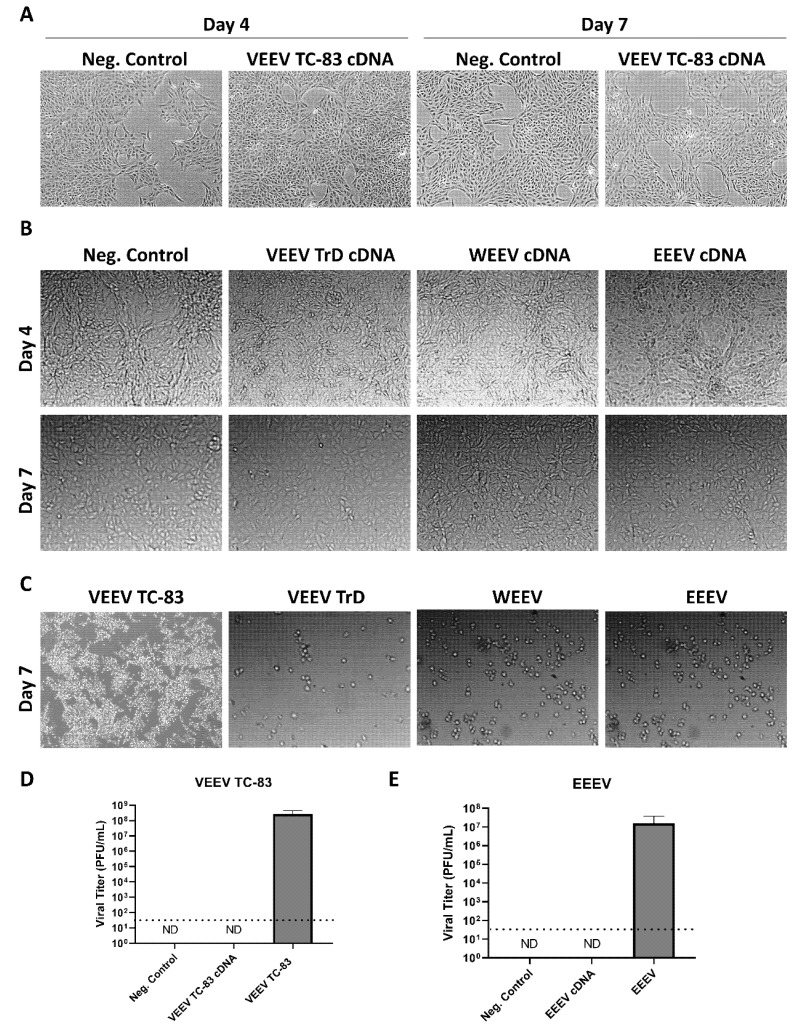
cDNA synthesis followed by RNase treatment renders VEEV, EEEV and WEEV RNA non-infectious; (**A**,**B**) RNA isolated from uninfected Vero cells (neg. control), VEEV TC-83, VEEV TrD WEEV, or EEEV infected Vero cells was converted to cDNA and treated with RNase A and H. cDNA samples were then transfected into Vero cells. Images were taken at 4- and 7-days post-transfection; (**C**) Vero cells were infected with VEEV TC-83 (MOI 5), VEEV TrD (MOI 0.1), WEEV (MOI 0.1), or EEEV (MOI 0.1) as positive controls for CPE. Images were taken at 7-days post-transfection. Results shown are representative of two separate experiments. (**D**,**E**) Plaque assays were performed on Day 7 supernatants from VEEV TC-83 and EEEV cDNA transfected cells. Supernatants collected at 24 h p.i. from VEEV TC-83 and EEEV infected cells (MOI 5) were included as a positive control. ND = none detected. The dotted line indicates the limit of detection.

Another common method of processing RNA is converting it to cDNA to enable quantification of gene expression via qPCR. Therefore, we next assessed whether conversion to cDNA rendered alphavirus RNA non-infectious. Following a cDNA synthesis reaction, samples were treated with RNase A and H to digest any remaining viral RNA that may initiate viral replication upon transection. RNase H is an endonuclease that cleaves RNA found in RNA-DNA duplexes, whereas RNase A is an endonuclease that cleaves pyrimidine-containing sites in single-stranded RNA [11]. Samples from VEEV TC-83 or VEEV TrD infected cells processed in this manner did not induce CPE at Days 4 or 7 (Figure 6A,B). They looked similar to the cells transfected with cDNA from uninfected cells (negative control), adherent and spread out, demonstrating the characteristic morphology of healthy growing cells. cDNA processing and RNase A and H treatment were also effective at inactivating viral RNA isolated from cells infected with the related encephalitic alphaviruses, EEEV and WEEV (Figure 6B). In contrast, Vero cells infected with VEEV TC-83, VEEV TrD, WEEV, and EEEV displayed dramatic CPE at Day 7, with floating, rounded and fragmented cells, characteristic of dying/dead cells (Figure 6C). To further confirm that cDNA synthesis followed by RNase treatment renders alphaviruses non-infectious, plaque assays were performed on Day 7 supernatants from VEEV TC-83 and EEEV cDNA transfected cells. No infectious virus was detected in these samples (Figure 6D,E). In contrast, positive control VEEV TC-83 and EEEV infected samples displayed an average infectious titer of 2.6 × 10^8^ and 1.6 × 10^7^ PFU/mL, respectively. These data indicate that conversion of alphavirus RNA to cDNA followed by RNase A and H treatment renders it non-infectious.

## 4. Discussion

This study demonstrated the inactivation of the infectious genome of VEEV using two methods, viral RNA fragmentation and cDNA synthesis. Previously, other researchers confirmed the ability of AVL buffer and TRIzol LS to render suspensions of positive-strand RNA viruses non-infectious to cells in tissue culture [12]. In these studies, AVL/TRIzol LS treated suspensions of positive-strand RNA viruses were directly added to Vero cells at various dilutions to confirm a lack of virion viability. To the best of our knowledge, no method has previously been published for the inactivation of the genomic RNA in positive-strand select agents. Also, previously, it has not been reported whether viral RNA is able to infect the cells in the absence of an introduction method such as transfection. In our studies reported here, we used a TRI reagent to disrupt viral and cellular membranes and isolate RNA, which was then used for fragmentation or addition experiments that are important for positive strand RNA virus studies. We confirmed the inactivation of the viral RNA post-fragmentation by monitoring for CPE in the cells transfected with the fragmented viral genome. We did not observe any cytopathic effect in these cells 13 days post-transfection, while the positive control showed vast CPE at day 3 post-transfection. We also confirmed the inability of fragmented RNA to produce infectious virions by performing plaque assays using the supernatant from the cells transfected with fragmented viral RNA, and we observed no plaques in the cells treated with this supernatant. In contrast our positive control showed plaques with average viral concentration of 7 × 10^8^ PFU/mL.

We demonstrated the inability of viral RNA to enter cells in the absence of transfection by directly adding purified viral RNA to Vero E6 cells and assessing cell death. The inability of viral RNA to enter the cells in the absence of transfection was further confirmed by assessing the presence of viral capsid and nsP1 sequences inside these cells using qRT-PCR. The qRT-PCR did not detect viral nsP1 sequence in the samples that received the viral RNA in their media, while the viral capsid sequence was detected at Ct values > 36 in these cells. The viral RNA was detected at Ct values < 16.3 in the positive control (Figure S3). As the higher Ct value correlates with a lower copy number of the RNA in the sample, our result suggests a low number of viral genomes in the cells that received viral RNA in their media in the absence of transfection. VEEV is known to replicate rapidly in infected cells, and the presence of virus in the supernatant was reported as early as 6 h p.i., with the peak of the virus release occurring at 24 h p.i. [13]. We showed that transfection of concentrations as low as 5 ng of viral RNA could cause CPE, suggesting the initiation of the viral life cycle and infectious virion formation, resulting in CPE at 72 h post-transfection. As our results did not show any CPE in Vero E6 cells after adding viral RNA directly to cell monolayers (in the absence of transfection) for 13 days post-treatment, we speculate that the viral genome detected in our qRT-PCR experiments is the remainder of the viral RNA outside of the cells, and the purified viral genome is non-infectious unless deliberately introduced into cells in vitro (via transfection or other methods of introduction to the cells). Considering that we used only one cell type in our experiments, further in vitro and in vivo experiments are needed to confirm this result.

We also found that converting RNA from virally infected cells to cDNA coupled with RNase A and H treatments, rendered VEEV, EEEV, and WEEV RNA non-infectious. It is important to note that EEEV is a select agent pathogen, whereas WEEV is not. However, both viruses must be handled in a BSL-3 laboratory. Thus, the viral genome of the positive-strand RNA viruses is not likely to be a direct hazard to laboratory workers but can produce infectious viruses if it is introduced to susceptible cells. This raises a concern regarding the production of highly contagious viruses outside of authorized laboratories, which is particularly important for select agents. Thus, the further inactivation of genomic viral RNA is necessary for select agents with positive-strand RNA genomes before removal from an authorized laboratory. Here we report two protocols for performing viral RNA inactivation to address these concerns.

## Figures and Tables

**Table 1:** The sequences of the primers and probes used to detect VEEV TC-83 in Vero E6 cells.

Primer and Probes	Sequence
Capsid FWD	GGACGACCCATTCTGGATAAC
Capsid REV	CGTTCCACATGACGACTGAA
Capsid Probe	/5SUN/TCCTTCATT/ZEN/CACACCTCCCAGCAC/3IABkFQ/
nsP1 FWD	CTGACCTGGAAACTGAGACTATG
nsP1 REV	GGCGACTCTAACTCCCTTATTG
nsP1 Probe	/56FAM/TCCGTCAAC/ZEN/CGCGTATACATCCTG/3IABkFQ

## Supplementary Materials

The following are available online at https://www.mdpi.com/article/10.3390/v14020272/s1, Figure S1: Viral RNA does not cause CPE without artificial entry; Figure S2: Viral RNA does not cause CPE without artificial entry; Figure S3: VEEV TC-83 capsid and nsP1 gene detection in cells treated by adding VEEV TC-83 RNA to their media.
