# Supplementary material for: Inactivation of Venezuelan Equine Encephalitis Virus Genome Using Two Methods
Source: Viruses. 2022 Jan 28;14(2):272. doi: 10.3390/v14020272 (PMC8878209; doi:10.3390/v14020272)
Supplement: Supplementary file 1 [file viruses-14-00272-s001.zip › viruses-1576369-supplementary.pdf]

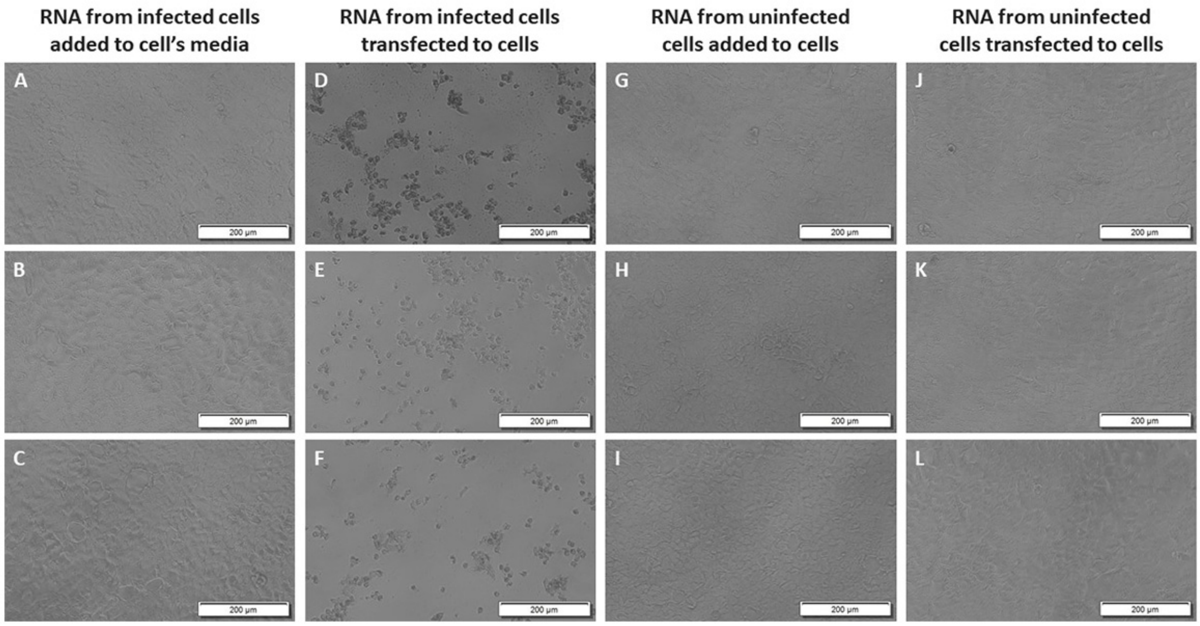

Figure S1. Viral RNA does not cause CPE without artificial entry. Total RNA purified from uninfected or TC-83 infected Vero cells directly added to media or transfected to Vero cells, images taken at 7d post-administration; (A-C) 1ug/well of total RNA from TC-83 infected Vero cells directly added to Vero cells; (D-F) 1ug/well of RNA from TC-83 infected Vero cells transfected to Vero cells (positive control); (G-I) 1ug/well of total RNA from uninfected Vero cells directly added to Vero cells (negative control); (J-L) 1ug/well of total RNA from uninfected Vero cells Transfected to Vero cells (negative control).

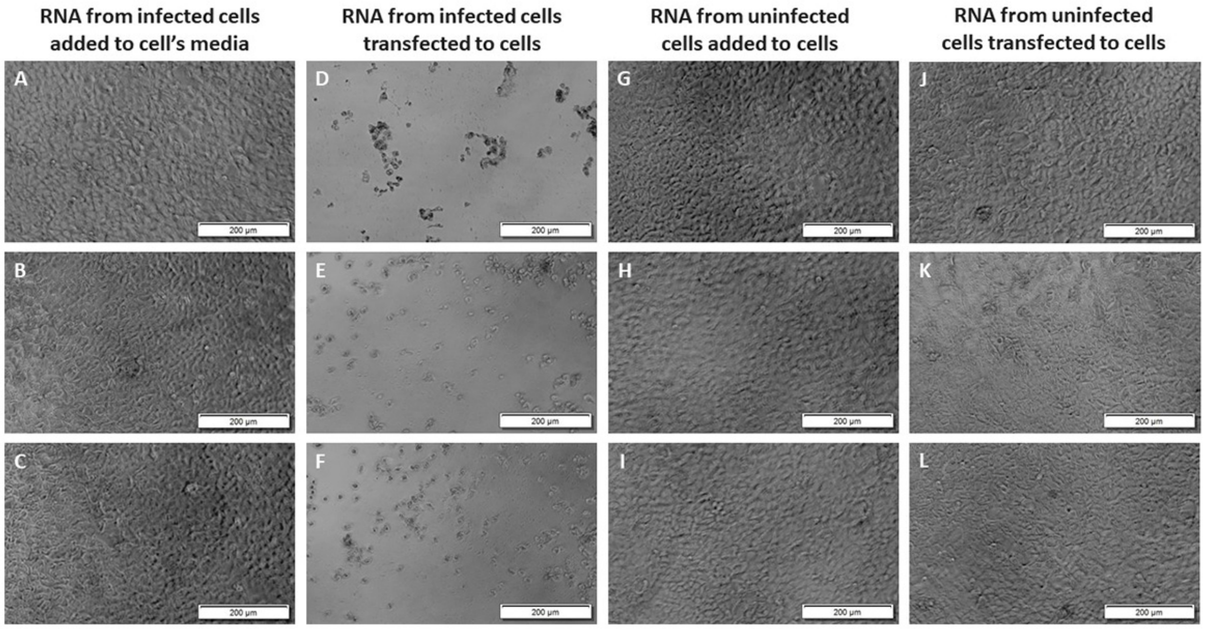

Figure S2. Viral RNA does not cause CPE without artificial entry. Total RNA purified from uninfected or TC-83 infected Vero cells directly added to the media or transfected to Vero cells, images taken at 13d post-administration. (A-C) 1ug/well of total RNA from TC-83 infected Vero cells directly added to Vero cells. (D-F) 1ug/well of RNA from TC-83 infected Vero cells transfected to Vero cells (positive control). (G-I) 1ug/well of total RNA from uninfected Vero cells directly added to Vero cells (negative control). (J-L) 1ug/well of total RNA from uninfected Vero cells Transfected to Vero cells (negative control).

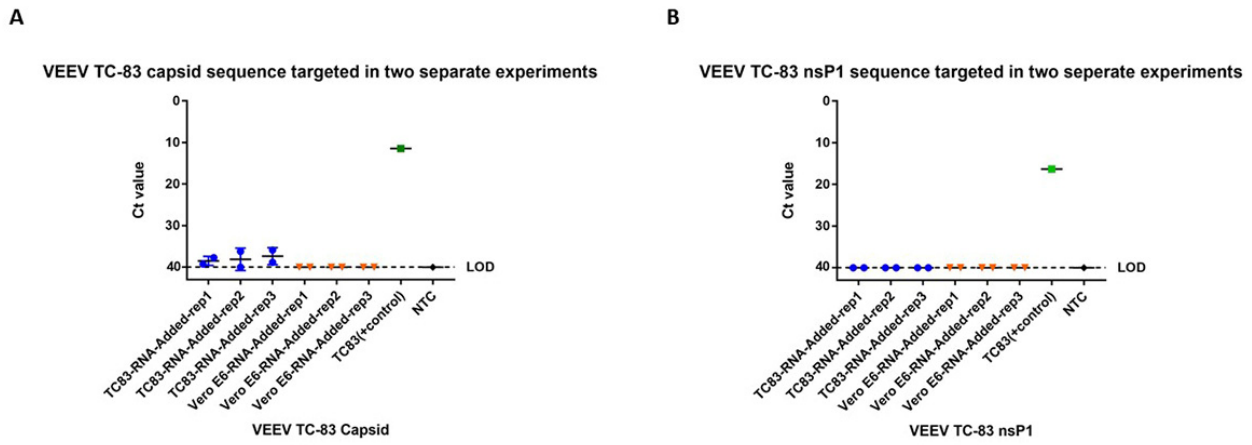

Figure S3. VEEV TC-83 capsid and nsP1 gene detection in cells treated by adding VEEV TC-83 RNA to their media. 10ug/well of total RNA purified from VEEV TC-83-infected cells was directly added to Vero E6 cell media in triplicate. As a control, 10ug/well of total RNA purified from uninfected Vero E6 cells was directly added to the Vero E6 cells in triplicates. Total RNA from VEEV TC-83 infected Vero E6 cells (5MOI) was used as positive control. RNA was extracted from all groups at 72h post-treatment, and 500ng of each RNA sample was used for cDNA synthesis. Synthesized cDNAs were diluted 1:100 and used for qPCR (2ul/rxn). Three reactions per sample cDNA was performed. Each point in the graph represents the mean of three qPCR reactions per experiment. The colors in the graph represents different treatment groups as follows: (Blue) samples treated with adding VEEV TC-83 RNA to their media, (Orange) samples received uninfected Vero E6 RNA in their media, (Green) VEEV TC-83-infected Vero E6 cells, positive control (shown in average of triplicates), (Black) non-template control(NTC). (a) VEEV TC-83 capsid sequence targeted in qPCR. Viral capsid detected at Ct values >36 in the cells treated with 10ug/well total RNA from VEEV TC-83-infected Vero E6 cells. The Ct value for the positive control is <11, and for cells treated with total RNA from un-infected Vero E6 cells and NTC Ct was undetected. (b) VEEV TC-83 nsP1 sequence targeted in qPCR. Viral nsp1 was not detected in the cells treated with 10ug/well total RNA from VEEV TC-83-infected Vero E6 cells. The Ct value for the positive control was <16.3, and for cells treated with total RNA from un-infected Vero E6 cells and NTC was undetected.
